# The impact of the COVID-19 pandemic on the experiences of Nova Scotian dentists

**DOI:** 10.1177/10519815251351600

**Published:** 2025-06-26

**Authors:** Katherine Culligan, Christina Holmes, Arlinda Ruco

**Affiliations:** 1 Interdisciplinary Health Program, St. Francis Xavier University, Antigonish, NS, Canada; 2 Peter Gilgan Centre for Women's Cancers, Women's College Hospital, Toronto, ON, Canada; 3 Beatrice Hunter Cancer Research Institute, Halifax, NS, Canada; 4 Nova Scotia Health, Halifax, NS, Canada; 5 Research and Innovation, VHA Home HealthCare, Toronto, ON, Canada

**Keywords:** dentistry, Nova Scotia, COVID-19, pandemic, motivation to work

## Abstract

**Background:**

The COVID-19 pandemic has significantly impacted how healthcare practitioners operate and provide care. Dentists are at an increasing risk of contracting the disease; working in mouths and constantly exposed to patient respiratory droplets and aerosols.

**Objective:**

We explored the experiences of Nova Scotian dentists including motivation to work and perceived organizational support during a global public health crisis.

**Methods:**

This was a mixed-methods study utilizing a sequential exploratory design. Phase 1 involved qualitative interviews with eight Nova Scotian dentists regarding their pandemic experiences. In Phase 2, a cross-sectional survey informed by Phase 1 focused on motivation to work and perceived organizational support. Descriptive statistics summarized the study sample, while Chi-square and t-tests explored associations and differences between subgroups.

**Results:**

Phase 1 revealed logistical and physical challenges, including difficulty obtaining and working in personal protective equipment. Barriers to motivation included financial stresses and concern about patients without care. A key recommendation was against complete practice shut down in future pandemics. Clear return to work plans provided by the Nova Scotia Dental Association (NSDA) were identified as important support. Phase 2 revealed a high level of perceived organizational and regulatory body support among respondents (n = 19). Men exhibited slightly higher intrinsic motivation compared to women. Changes in patient needs included clenching and grinding, (the “Covid clench”), as top observations by dentists.

**Conclusions:**

This study's insights into dentists’ occupational challenges suggest that regulatory guidelines helped streamline return-to-work, and future improvements in crisis preparedness and business management would be beneficial.

## Introduction

There has been a great deal of research on front-line healthcare workers during the COVID-19 pandemic, but dentists are one group that has been overlooked.^1–3^ For example, in a recent scoping review of healthcare workers’ experiences during the pandemic, none of the 161 included studies focused on the experiences of dental practitioners specifically.^2^ Dentists provide essential oral health services and there is strong evidence showing that oral health plays a large role in the overall health of individuals, especially in older ages.^4^ Dentists are also at an increased risk of contracting COVID-19 due to the nature of their work, working in mouths, and constantly being exposed to patient respiratory droplets and aerosols.^5^ In Nova Scotia, Canada, the provincial government ordered a complete shut-down of almost all dental offices in the province (except 8) for two months at the beginning of the pandemic.^6^ The 8 practices that remained open under strict protocols were limited to patients that required emergency dental care.

Recent literature has emerged in several countries assessing dentists’ experiences during the COVID-19 pandemic. Common among these studies is the high levels of stress and anxiety reported by dentists during the pandemic.^7–10^ The increased stress and anxiety are likely influenced by several factors including concern for their own health and well-being^8,9,11^; availability of PPE and changes to treatment protocols^7,9,11–13^; financial strain and long-term impacts on dental practices^7–9,14^; and changes to patient oral health.^9^ No research to date however has assessed the experiences of Canadian dentists.

The Hackman and Oldham Job Characteristics Model states that positive work outcomes such as high internal motivation, quality work performance, employee satisfaction, low absenteeism and turnover are influenced by five key core dimensions of one's job including skill variety, task identity, task significance, autonomy, and feedback.^15^ When a stressor, such as the COVID-19 public health crisis impacts one's working conditions, it can lead to changes in these key outcomes like satisfaction and motivation. Recently, there has been an increase in the number of studies focused on work motivation and productivity in the context of the COVID-19 pandemic.^16^ Given how the pandemic has impacted so many facets of dentists’ practice including skill variety (e.g., performing new tasks such as virtual consultations) and autonomy (e.g., mandated infection prevention protocols), it is important to explore how dentists’ experiences during the pandemic may inform their motivation to work and their willingness to provide high quality patient care. From the perspective of employers and population healthcare, it is important to also understand what organizational supports are needed to maximize employee motivation and ensure that dentists remain in the profession. Given that future public health crises are unavoidable, it is imperative to better understand how work stressors such as a pandemic may impact healthcare professionals’ motivation and job satisfaction.

Therefore, the objective of our study was to explore the experiences of Nova Scotian dentists during the COVID-19 pandemic with a specific focus on dentists’ motivation to work during this crisis.

## Methods

### Study design and setting

This was a mixed-methods study using a sequential exploratory design^17^ conducted in Nova Scotia, Canada. Participants eligible for our study included general dentists that had practiced in Nova Scotia for at least six months during the COVID-19 pandemic, starting in March of 2020. Dentists that were no longer practicing were still eligible to participate if they had practiced for at least 6 months during said timeframe.

### Phase 1

A qualitative descriptive approach was used to gather in-depth insights into participants’ experiences and perceptions. Snowball sampling was utilized to recruit participants starting out by initially emailing dentists with whom the study team was already familiar. We also ensured that we had representation from dentists practicing in both rural and urban settings to gather a range of perspectives, using targeted sampling to contact dental practices in desired areas directly. Eight dentists agreed to participate in this phase of the study and data was collected from July to August 2023.

We collected data using semi-structured interviews which spanned from 25–70 min. Interviews were completed in-person, or through online video calls, via Zoom. An interview guide with open-ended questions and prompts to facilitate discussion was used (Appendix 1). The guide focused on participants’ experience as a dentist, their work during the COVID-19 pandemic and how it changed, concerns/challenges they experienced, and the overall impact of the pandemic on patient care, oral health overall, and the field of dentistry.

All interviews were recorded and transcribed verbatim via OtterAI, a transcription software, and edited, to ensure accurate representation of participants’ narratives and enable a subsequent analysis of the qualitative data. A deductive approach was mainly taken and was mixed with an inductive approach using thematic analysis to analyze the data. With deductive coding, we identified four categories for analysis: administration, motivation to work, patient themes, and future applications or recommendations. Using this framework, we went through each transcript and identified data representative of these categories within the transcripts but also allowed for new codes (inductive coding) to emerge through identifying patterns and themes organically from the data. This allowed for a comprehensive overview of the participants’ experiences. The qualitative analysis was facilitated by NVivo Software (version 14.23.0) which aided in organizing and managing the dataset. To ensure rigour, a second researcher on our team coded a random sample of transcripts to ensure good inter-coder reliability.

### Phase 2

A cross-sectional anonymous survey was employed in Phase 2 to further explore certain findings from Phase 1 that were identified as important or needing to be expanded upon. Participants that completed Phase 1 were also eligible to complete Phase 2. A population sampling approach was used, and recruitment of participants was done through the dissemination of the survey by the Nova Scotia Dental Association (NSDA) to their membership. It is estimated that there are approximately 576 dentists practicing in the province.^18^ The link to the study survey was shared via email and in the NSDA's bi-weekly newsletter. Reminders were sent out at two and four weeks after the initial invitation. We also sent out the survey link to all registered individual dental practices in the province as identified through the Dentist Directory of Canada, an online registry. Data for phase 2 was collected from October 2023 to February 2024.

The survey instrument was administered through Qualtrics (version February 2024) and included a total of 17 items focused on demographics, organizational support, motivation to work, and observations related to patient care. Participants completed the following validated instruments as part of the survey including the COVID-19 organizational support scale (COVID-OS)^19^ and the Perceived Organizational Support (POS) scale.^20^ Participants also completed the Motivation at Work Scale (MAWS),^21^ a validated instrument used to determine what reasons a worker has for doing their job. Questions on the MAWS map onto one of four different types of motivation at work including intrinsic motivation, identified regulation, introjected regulation, and external regulation.^21^ All three validated instruments utilized 7-point Likert scales and were adapted to fit our study context by making some of the language fit the target professional group for this study. Other items related to oral health concerns and several open-ended questions were also asked to offer space for participants to elaborate on some of their answers. Upon completion of the survey, participants had the option to proceed to an external and separate link where they could input their email to be entered in a raffle to win an iPad.

Descriptive statistics were used to summarize the study sample. Analysis of Likert-scale questions involved calculating weighted averages to summarize participants’ responses. For items that were positively framed, a higher score was deemed as better versus items that were negatively framed and therefore reverse scored, where a lower mean was interpreted as having a better outcome (e.g., higher motivation). Chi-square tests were used to explore associations between categorical variables and independent samples t-tests were run to determine if there were statistically significant differences by gender, years of practice, and practice location. All statistical analyses were conducted using the Statistical Package for the Social Sciences software (SPSS), version 28.0.1.1 (14).

## Results

### Phase 1

We interviewed 8 participants in Phase 1 including 4 men and 4 women. 4 dentists were practicing in urban versus rural areas. Four dentists had over twenty years of practice, three had ten to twenty years of practice, and one had five to ten years of practice (see Table 1). All 8 dentists interviewed had been practicing in Nova Scotia prior to the initial COVID-19-related shutdowns in March 2020 and continued practicing through to the time of their interview in 2023. As such, participants had practiced through multiple phases of the pandemic, including both its early and later stages.

**Table 1.** Qualitative participant demographics.

		*N (%)*
Location of dentistry practice	Rural	4 (0.5%)
	Urban	4 (0.5%)
Years of practice as a dentist	0– < 5	0 (0.0%)
	5– < 10	1 (0.13%)
	10– < 20	3 (0.38%)
	20+	4 (0.5%)
Gender	Women	4 (0.5%)
	Men	4 (0.5%)
Practice Ownership	Private	7 (0.88%)
	Corporate	1 (0.13%)
Salaried dentist	Previously, pre-COVID-19	1 (0.13%)
	No	7 (0.88%)

Tables 2 and 3 present summaries of the key themes alongside definitions identified from the Phase 1 data. These themes were informed by the deductive coding structure drawn from the literature on motivation to work and health professional challenges with COVID-19 as well as the inductive coding approach.

**Table 2.** Dentists’ challenges, motivation to work, and patient observation themes from interviews on dentists’ experiences during the COVID-19 pandemic.

Theme	Subtheme	Description	Example
1. Challenges	a. Physical	Statements regarding issues that arose from physical changes to the dentist office and how dentists were practicing such as struggles with the extra PPE.	*“We had to wear gowns, and then there was the face mask on top of that. It made things hard as far as visibility…the face mask gets in the way and there is a glare which can distort our vision a bit.”* “*The extra PPE was very uncomfortable [and caused] overheating for us [as dentists].”*
	b. Logistical	Statements about any challenges that arose related to practice management such as sourcing of PPE and change of care administration.	*“Everyone paid three times the cost of what it would usually cost for a box of like gloves or masks. I found that outrageous.”*
2. Motivation to work	a. Barriers to motivation	Statements of any factors that hindered dentists’ motivation to do their job, stressors such as financial struggles.	*“I had no income coming in, so I basically floated the practice for those three and a bit months… I will honestly say it was a terrifying time.”*
	b. Supports to motivation	Statements of any factors that increased dentists’ motivation to do their job such as colleague interactions.	*“When we came back, we were quite busy, so we were happy to be back and seeing patients. It was good for us to be making money again, after not making money for the past three months.”*
3. Patient observations	a. Patient attitudes	Statements that refer to patient behaviour and how this may have changed during practice closures.	*“[patients would say] why do I have to wear a mask just to walk in [the clinic] I’m going to take it off. And it's like, are you kidding me?”*
	b. Oral health changes	Statements that speak to any observed changes in patient dental health.	*“We saw a lot more people requiring bite plates and more people were clenching and grinding with the stress so there were a lot more broken teeth. A lot more cavities as well with people eating more when they were at home that they wouldn’t have eaten before.”*
	c. Return to care	Statements discussing patient health seeking behaviours, or hesitancies to seek care once practices were open.	*“I think a few people had reservations to come into the dental office, especially when you’re drilling into mouths with the aerosols when it was still new.”* “*For the patients I did see, I think people felt quite safe, like patients would say to me, I feel safer coming to your office than I do going to the grocery store. Because I think we did a really good job communicating to our patients our protocols, and what we were doing to keep them safe.”*

**Table 3.** Dentists recommendation themes from interviews following dentists’ experiences with the COVID-19 pandemic.

Theme	Subtheme	Description	Example
4. Future recommendations	a. Practice management	Statements that speak to the need for additional training in the business side of practice management.	*“When the first round of that funding came out from the provincial government about small businesses, we were not allowed to access that. So all of a sudden, I’m not a small business that's worthy of that?”* *“I mean, when you’re in dental school, they’ll tell you that being a dentist is the easiest part. It's all the other stuff that grinds you down a bit. There is definitely lots of that post pandemic.”*
	b. Pandemic insurance	Statements that suggest awareness of insurance options are crucial.	*“So [X company] had a clause in their business insurance, that if there was a pandemic that they would pay out basically. So I like to call this ‘pando-payout’. A lot of the dentists received ‘pando-payout’ because they were with [X]. [X]is also one of the more expensive ones. And I wasn't with them … I went with a local company for my insurance. And lo and behold, they did not have pandemic payout.”*
	c. Regulatory bodies	Statements that examine the response from the dental regulatory bodies in the province and suggestions for future support.	*“, the Dental Board, I think did a fantastic job coming up with a return-to-work plan, with very clear instructions of what they expected from us as practice owners about how we were going to implement these plans”* “*I think the government did pretty well but if there were another pandemic, I hope we would not have to be closed like that because we learned a lot… the preparedness and knowing how to set up the dental office for a case of a pandemic, things like that.”* “*So I want to see … more of stepping up to the plate, like obviously, not putting yourself in undue harm's way. But stepping up to the plate, fully protected from exposure and whatnot. But prepared to treat patients, especially patients who were having an emergency, being able to treat that in your clinic, as opposed to sending them far away to access substandard care.”*
	d. Community & Practice Building	Statements that discussed the importance of building community with other dentists or within practices in order to cope with the pandemic	*“And we workshopped that document together so that we could like rather than me just saying top down, ‘here's what we're doing everybody abide by it’. I wanted to make sure that everybody felt that they had a say in what their return-to-work safety environment looked like. And I still think that was the best thing we could have done.* “*[T]here was some good community building that came out of [the pandemic], that NSDA helped with. They did an online webinar … mental health support for employers in the dental field and I think they said it was the one of the most historically well attended webinars”*

#### Theme one: challenges

Dentists were faced with multiple challenges upon closure of their practices, but even more so faced challenges once they were able to reopen. Physical challenges and logistical challenges were identified as key subthemes as these acted as barriers for dentists to be able to operate and provide a high standard of care to their patients.

During lockdown, emergency offices operated at limited capacity, but dental care was limited to patient calls in practices that were shut down, completely changing dentists’ care provision and nature of work: “*And all we could do was prescribe; we’d be on the phone, prescribing people antibiotics, and I have never called in so many prescriptions in my career”* (Participant #03)*.*

Upon return, dentists had to adapt to a one-patient-per-room approach which required operational shifts within practice layouts, sometimes creating a whole new space: “*And for the most part, you almost have to create another sterile room”* (Participant #08). Many changes required lasting scheduling shifts, such as accommodating for sanitation time and COVID-19 screenings. Dentists had no choice but to comply if they wished to return to practice, but these changes came at high costs:

[A]ll these sterilization instruments and trays and they take more room, take more time, we’ve had to hire more people, like we actually have a designated sterilizer. … And obviously, still to this day, that's an added cost. Sterilization [is] not something that you recover, you can’t bill for sterilization. Patients need everything sterilized anyways, and this has created lasting changes” (Participant #08).

They also had to obtain equipment such as ventilation units and new laundry machines to meet safety requirements, even hiring a new staff member that would be solely responsible for the laundry: “*[W]e ended up having to hire, you know, what we call a float, like somebody that can just help keep the laundry going.”*

Dentists also faced difficulties in sourcing personal protective equipment (PPE) upon return. With the PPE provided being of poor quality, some dentists bought disposable gowns or even sewed their own gowns, but the majority of PPE was outsourced at astronomical costs in comparison to pre-pandemic times*: “[T]he costs were crazy. [A] box of gloves that pre-pandemic had been under $20. … At the peak, you were paying $130 for a box of gloves, and on average, you’re going through an entire box of gloves a day”* (Participant #03).

Physical challenges also arose with the PPE that dentists were able to get a hold of during the pandemic, as it interfered with the physical ability to carry out their critical dental tasks, adding heat, weight, and other complications. Dentists even remarked that they could not breathe very well with the required level of masks: “*… [T]hat was a challenge, literally just seeing and having a mask on and a level three or a fitted mask. Because they’re just harder to breathe”* (Participant #08). Seeing also arose as an issue with the added equipment, as face shields did not fit over loops that dentists use to see: “*The face shield I found very annoying because it doesn’t fit well over your loops. So, you couldn’t really see as well, to be honest, which is when you’re doing fillings or you know, you really want to be able to see”* (Participant #02).

#### Theme two: motivation to work

Dentists faced various barriers to motivation with a major barrier being the many stresses that arose as a result of the COVID-19 pandemic. This was a major theme discussed at length in interviews. These stresses included the unknowns surrounding the pandemic which caused uncertainty as well as concerns of being able to provide care for patients:

“I was really nervous coming back because I didn’t know what everything was going to feel like. We were having to wear gowns and face shields, and we didn’t know how that was going to work with providing treatment to patients. So, it was really the unknown that made me a little anxious coming back” (Participant #01).

Financial stress was one of the largest barriers to motivation, and many dentists expressed the struggles they faced financially with practice management in keeping their practice afloat:

“In my head, I’m thinking, I don’t even know if we are coming back. I don’t know if I might lose my business over this. I can remember having supper with my husband and having multiple conversations about bankruptcy. I’ve never used that word in my life. But that's where we were” (Participant #03).

This was compounded with the stress of the responsibilities that come with leading a dental team:

“[T]he underlying conversation with every person I talked to was, I just don’t love it anymore. It's just that everything is such an effort now. And many of us haven’t paid ourselves in months. So, if you’re volunteering your time to keep everybody else employed, and everybody else healthy and safe… how sustainable is that? You can do that for a while but after three years, it gets really hard on the head” (Participant #03).

On the other hand, participants expressed areas that supported motivation. One support was their eagerness to return to practice as that would mean they had incoming business and would also benefit from the positive interactions and collaboration with co-workers: “*I think [the stress] was more offset by the joy of coming back to work and the social aspect of seeing each other, seeing patients, seeing the mail person again and things like that”* (Participant #07).

Dentists also commented that regulatory decisions and responses were communicated effectively and helped organizationally to streamline dental practice and alleviate obstacles, which in turn also enhanced motivation: *“I think that was one thing that the NSDA got right. [T]hey were monitoring other provinces and seeing what other provinces were doing”* (Participant #07).

The pandemic changed the ways that dentists do their work, which ultimately shifted motivation, resulting in some supports from their environment, but many more barriers, such as stress of the unknown, financial stress, and the stress of leading a dental team. It is important to note that dentists were also concerned for their patients, and barriers to motivation included the stress they felt in not being able to provide for their patients. This in turn, brought many interviews to the topic of patient observations, pre, and post-pandemic.

#### Theme three: changing patient needs & patient attitudes

Patient care was a crucial concern of the dentists interviewed. An unanticipated theme that arose was that dentists saw major physical changes in oral health after the pandemic such as clenching, grinding, and broken teeth. They explained that many of these were likely due to stress: “*Stress, stress, stress, stress, stress. [It's] very interrelated, you know, stress, there's clenching, grinding, that creates, you know, stress and strain on the teeth and can create sensitivity”* (Participant #03). Some dentists used the term the “Covid clench” to refer to the stress-related clenching they had observed. Dentists mentioned these observed oral health changes as never seen before in their career:

“[A] lot of broken teeth, a lot of clenching and grinding related injuries. People's stress levels continue to be through the roof…historic in anything I’ve seen for almost 20 years that I’ve been doing this. People breaking their dentures, people breaking the night guards in half because they’re clenching so hard” (Participant #03).

Patients’ attitudes related to pandemic measures tended to be positive and they were generally compliant with dental recommendations. There were, however, few exceptions, in which dentists did their best to be strong against pushback, but which also highlight the unique context of dentistry, related to mask mandates:

“[O]nce we started understanding more about COVID and there [was] more and more pushback. The pushback about the masking was just … I think all of us are just so done with fighting with patients, but [patients would say] why do I have to wear a mask just to walk in [the clinic] I’m going to take it off and it's like, are you kidding me?”

#### Theme four: future recommendations

Dentists were asked questions on pain points from the profession that were exposed during the pandemic, as well as lessons learned and recommendations for the future. They offered insights on gaps in training such as in practice management but also remarked that regulatory bodies were successful in their support, but that it was mainly reactive. Suggestions for the future were to avoid complete shutdown and to be recognized as essential services. Participants emphasized practice management as a large issue within dentistry that was exposed during the pandemic. They remarked that dentists need training on managing the business aspect of their practice, so as to be able to tackle many administrative challenges that are often not acknowledged: “*The running a business aspect was not really something I thought about a lot before going into dentistry[.] I would say you don’t really appreciate how much work running a small business is, and COVID certainly highlighted the negative aspects”* (Participant #02). Dentists mentioned that they were told in school that the dental work is the easiest part of the job, and that they soon realized the truth of this statement: “*I mean, when you’re in dental school, they’ll tell you that being a dentist is the easiest part. It's all the other stuff that grinds you down a bit. There is definitely lots of that post pandemic”* (Participant #06).

Additionally, awareness of insurance options for a dental practice, including pandemic insurance, is crucial. Some practices faced difficulties in claiming pandemic insurance, revealing a need for better understanding and more inclusive requirements for eligibility. Other dentists ended up selling their practices and transitioning to a corporate ownership style post-pandemic, as the business management became too much for them:

“They’re selling [their practices], even though they don’t really want to sell now, but they sort of want to get out of the business side of dentistry [so] they end up selling to big companies. And that's sort of the lasting impact that I’m seeing from COVID” (Participant #07).

It is important to note that many dentists had observed the shift from privately-owned practices to corporately owned practices in the past many years, but that COVID had encouraged this shift to occur at a much faster rate.

Finally, participants looked back upon their experiences practicing through the COVID-19 pandemic, with many reflections offered on what was successful as well as recommendations in the event of a future pandemic. Many participants emphasized the need for clear communication by both employers and regulatory bodies. Regulatory bodies were successful at providing comprehensive documents laying out restrictions and return-to-practice recommendations and this was recommended in the future:

“When we came back again, the Dental Board, I think did a fantastic job coming up with a return-to-work plan, with very clear instructions of what they expected from us as practice owners about how we were going to implement these plans. And it was a document” (Participant #03).

Online resources such as virtual meetings, conferences, and online forms also helped in keeping dentists connected and informed:

“[T]here was some good community building that came out of [the pandemic], that NSDA helped with. They did an online webinar … mental health support for employers in the dental field and I think they said it was the one of the most historically well attended webinars that they ever had. It just went to show how many of us were feeling so vulnerable as employers” (Participant #03).

The main takeaway from interviews was to look to the future, and to see what lessons dentists extracted from this epochal experience. In the future, participants discussed that they would like to see dental practices remaining open during pandemics, avoiding complete shutdowns to ensure continuous patient care:

“I think moving forward, things might be a little bit more of a system and more definitive. I think if a pandemic happened again, I like to think we would not be closed. I’m hoping that because of going through it, our association and our government would maybe have fine-tuned and streamlined things” (Participant #06).

### Phase 2

A total of 19 respondents were eligible for and completed the survey including 6 men and 13 women. This represents a response rate of 3.3%. The most common type of practice ownership was private (65%), and 35% of our sample had 20 + years of experience. Six dentists (30%) were salaried. When asked about the number of staff that worked in their dentistry practice, almost half of the sample (40%) said they worked in a setting with 20 + staff. Chi-square analyses revealed no differences in demographic variables by gender, years of practice, and practice location (data not shown).

When asked on a scale of 1–10 how supported dentists felt by their practice and professional association, the average score was 7.74 and 7.68 respectively. The finding of high perceived organizational support was also supported by the two validated instruments, the COVID-OS scale (data not shown) and the P-OS scale (data not shown). Briefly, the highest score on the COVID-OS scale was 5.96 for items related to having the ability to get tested for COVID-19 rapidly if they needed to, and the lowest score was 2.82 for items related to having access to childcare during increased work hours and school closures. The highest and lowest scored items were similar among men and women with no significant differences by gender, dentistry practice location (urban vs. rural) or salaried status found (data not shown).

Table 4 presents the scores for the MAWS stratified by gender. Participants had the highest mean scores for items related to intrinsic motivation followed by identified regulation, external regulation, and introjected regulation. The highest mean scores for women were items related to identified regulation and intrinsic motivation for men. Scores on the MAWS scale did not significantly differ by gender, practice location or salaried status. Open-ended questions revealed that dentists reported increased stress and exhaustion due to managing staff safety, financial concerns, and additional protocols, alongside financial strain, uncertainty, and challenges adapting to new procedures and regulations. Some also mentioned changes in career plans, with aspirations for certain specialties fading, such as oral-maxillofacial surgery, while others persisted in their work despite adversities.

**Table 4.** The MAWS scale stratified by gender (n = 19).

		*Men* *Mean* *(SD)*	*Women* *Mean* *(SD)*	*Total* *Mean* *(SD)*	*T-test* *P-value*
*Intrinsic Motivation*	Because I enjoy this work very much	6.33 (0.82)	5.08 (1.80)	5.47 (1.65)	0.13
	Because I have fun doing my job	6.17 (0.98)	5.00 (1.73)	5.37 (1.61)	0.15
	For the moments of pleasure that this job brings me	6.17 (0.98)	4.62 (1.85)	5.11 (1.76)	0.07
*Total Mean*				**5.32** (**1.65)**	
*Identified Regulation*	I chose this job because it allows me to reach my life goals	4.67 (2.07)	5.00 (1.29)	4.89 (1.52)	0.67
	Because this job fulfills my career plans	4.83 (2.04)	5.15 (1.35)	5.05 (1.55)	0.69
	Because this job fits my personal values	6.00 (1.10)	5.15 (1.21)	5.42 (1.22)	0.17
*Total Mean*				**5.12** (**1.43)**	
*Introjected Regulation*	Because I have to be the best in my job, I have to be a “winner”	3.00 (2.37)	3.15 (1.52)	3.11 (1.76)	0.87
	Because my work is my life and I don’t want to fail	2.33 (1.37)	2.77 (1.69)	2.63 (1.57)	0.59
	Because my reputation depends on it	3.83 (2.32)	3.15 (1.73)	3.37 (1.89)	0.48
*Total Mean*				**3.03** (**1.74)**	
*External Regulation*	Because this job affords me a certain standard of living	3.83 (1.94)	4.54 (1.45)	4.32 (1.60)	0.388
	Because it allows me to make a lot of money	3.00 (1.79)	3.69 (1.60)	3.47 (1.65)	0.41
	I do this job for the paycheck	1.83 (1.33)	2.54 (1.27)	2.32 (1.29)	0.28
*Total Mean*				**3.37** (**1.71)**	

Lastly, when asked if participants noticed a change in patients’ oral health-seeking behaviours during the COVID-19 pandemic, 100% answered yes. Responding to a question on what oral health concerns they noticed an increase in during the COVID-19 pandemic, 84% responded with grinding, or the “Covid clench”, tooth fractures (79%), gum disease (53%), hygiene related issues (42%), cavities (32%), and abscesses (26%). However, despite these observations, when asked on a scale of 1–10 what their level of concern was of the impact of COVID-19 on the oral health of their patients, the average score was only 5.84.

## Discussion

This study delved into the challenges and adaptations experienced by dentists during the COVID-19 pandemic. Our results suggest that dentists encountered significant physical and logistical obstacles, ranging from sourcing adequate PPE to redesigning practice layouts. Additional challenges included financial strain and enhanced sanitation protocols. Notably, dentists reported relatively high levels of organizational and professional support, particularly in terms of access to up-to-date information and recognition for their contributions. Despite facing uncertainties and stressors, dentists expressed resilience and identified intrinsic motivators, such as in-person interactions, as crucial for maintaining high levels of motivation. Moving forward, our study suggests that proactive measures and clear communication channels are important to navigating future crises effectively.

The results of our study are in alignment with prior research that shows that dentists were concerned with sourcing PPE, having good quality PPE, as well as other infection control protocols.^7,9,11–13,22^ Recently, a study by Prasetyo et al.^23^ explored the numerous factors influencing dentists’ job satisfaction during the COVID-19 pandemic. The authors also explored similar factors as in our study including perceived severity of the disease, staff cooperation & management commitment, PPE, stress, working hours, income, and overall satisfaction.^23^ In our study, perceived support from dentists’ professional association ranked similarly to perceived organizational support. Dentists felt they were valued for their contributions to the well-being of their practice, and that their practice showed concern for their wellbeing. Chevalier et al.^24^ found similar associations in their study on French dentists that saw organizational support as an important resource that improves job satisfaction of dentists.

Our analysis uncovered an interesting interplay between external stressors and drivers to motivation. Previous research shows the importance of actions like ensuring workplace comfort and enhancing employee well-being to counter COVID-19-induced anxiety and to boost motivation.^25^ Efforts to target motivation can lead to improved job performance, and so maintaining high job satisfaction is essential in organizations.^26^

Uncertainties surrounding the implemented regulations, lasting financial burdens, and the emotional toll of leading dental teams through unprecedented circumstances were identified as main stressors in our study. However, amidst these challenges, there were glimpses of resilience and support systems that bolstered motivation such as regulatory actions that allowed for streamlined approaches to the re-opening of practices. Most importantly, dentists talked about the in-person human interactions as a key motivator. This sentiment was supported by data from both phases where participants indicated highest mean scores for intrinsic motivation. This aligns with existing research stating that intrinsic motivation has a substantial impact on job satisfaction.^27^ Specifically, past research on dentists’ motivation has shown that the presence of intrinsic motivation has the most positive impact on the job satisfaction and well-being.^28,29^ Previous research has also shown that female university students were more extrinsically motivated, (which includes identified regulation), than their male counterparts.^30^ Given our limited sample size for Phase 2, we did not observe any significant differences in motivation by gender and there are no known studies observing gender differences in types of motivation amongst practicing dentists. This could be an important area for future research. Organizations may also want to consider how they support and motivate their dentists that are women, versus their dentists that are men.

Changing patient needs emerged as a central concern, with dentists observing notable shifts in oral health behaviours and conditions among their patients. This included teeth grinding, likely due to stress and referred to as the “Covid clench”, as the chief concern. This finding aligns with research on a Brazilian study sample, which showed that participants reported symptoms related to clenching and grinding at a high frequency during the pandemic.^31^ Additionally, these findings align with a recent study in the United States on dental care utilization and oral health during the pandemic which found a higher presence of psychological stress-related dental conditions.^32^

Finally, dentists offered insights into organizational support and regulatory decisions. They indicated that their professional association succeeded in supporting them as well as possible. This aligns with past research on the Nova Scotia Regulator of Dentistry and Dental Assisting (NSRDDA), formerly known as the Provincial Dental Board of Nova Scotia, (PDBNS), that stated that 78.8% of respondents believed their provincial dental board provided sufficient guidance during the pandemic.^22^ It is important to note that the NSRDDA is not the same as the NSDA as they are responsible for administering legislative decisions, while the NSDA is an organization that is member-based and aims to support dentists. These results brought to the forefront the need for proactive measures, clear communication channels, and collaborative efforts to navigate future crises effectively. As well, participants in Phase 1 highlighted concerns about dentists not being classified as essential services. It would be important to explore how dentists may continue to see patients in need in the event of a future pandemic.

### Implications

The findings of this study offer important implications for dental practices, professional organizations, policymakers, and researchers. Firstly, the challenges faced by dentists during the COVID-19 pandemic underscore the importance of proactive measures and adaptable practice strategies. The administrative burdens, logistical obstacles, and financial strains mentioned emphasize the need for support systems and clear communication channels. Moreover, the insights into dentists’ motivations emphasize the need for tailored strategies to boost morale. Such strategies may encourage the fostering of intrinsic motivation through the reduction of stress-inducing environments and behaviours. Other studies suggest that when environmental factors such as stressful interactions are controlled, intrinsic motivation can be increased.^33,34^

These implications resonate not only within the context of the pandemic but also in the broader landscape of crisis management and professional resilience. Dental practices can leverage these insights to enhance preparedness for future crises. Additionally, the importance of organizational support, regulatory clarity, and collaborative engagement should be emphasized. Professional associations and policymakers can use these findings to inform resource allocations to future curriculum and supports with a focus on crisis preparedness and business management training. Policymakers should consider dental practices as essential services, ensuring that dental professionals are adequately supported in times of crisis.

Furthermore, from a research perspective, this study contributes to the growing body of literature on the impact of pandemics on healthcare professions. By synthesizing qualitative narratives with quantitative assessments, this research offers a nuanced understanding of the challenges and adaptations experienced by dentists. Future studies in this area should continue to employ mixed method approaches to capture the multifaceted nature of professional experiences during crises.

### Limitations

While this study provides valuable insights into the experiences of dentists during the COVID-19 pandemic, several limitations should be acknowledged. First, the quantitative survey sample was rather small with a very low response rate. This significantly limits the representativeness and generalizability of these findings. Given such a low response rate, the results of this study may not be representative of the broader population of dentists in Nova Scotia and are not generalizable beyond those who completed the survey. However, we did have an opportunity to hear from a nearly even representation of men and women in Phase 1, as well as dentists from various regions of the province, both urban and rural. Phase 1 data collection also included rich in-depth accounts of participants’ experiences with similar accounts across multiple interviews. Additionally, the survey was circulated by the NSDA. As such, respondents may have been predominantly individuals who could have been reluctant to criticize the NSDA, potentially introducing self-selection sampling bias. Furthermore, the reliance on self-reported data and retrospective accounts may introduce recall bias and subjective interpretation. We also did not account for any temporal differences between the early and later stages of the pandemic where challenges and stressors amongst dentists may have varied. Participants’ perceptions of support, motivation, and patient impact may have been influenced by individual experiences and contextual factors, further limiting the generalizability of the findings.

### Future research

Building on the findings of this study, future research should explore additional factors influencing dentists’ experiences during crises, such as socio-economic disparities, professional identity, and institutional culture. It would be beneficial to look at the perspectives from patients, but also from the numerous professionals within dentistry outside of general practitioners, such as dental assistants, hygienists, and other dental specialties. Longitudinal studies could provide valuable insights into the long-term effects of the pandemic on dental practice and patient outcomes, allowing for the evaluation of resilience-building interventions and policy initiatives over time. Moreover, comparative studies across different healthcare professions could offer a broader understanding of the unique challenges and adaptations experienced by dental professionals relative to other healthcare providers. By identifying commonalities and differences across professions, future research can inform interdisciplinary approaches to crisis management and resilience building in healthcare settings.

## Conclusions

In conclusion, this study sheds light on the gaps in preparation and training within the dental profession, underscoring the imperative to integrate business management strategies. These strategies should be designed to harness intrinsic motivational factors, thus enhancing the meaningfulness of work, and bolstering internal work motivation. Moreover, there is a pressing need to advocate for regulatory bodies to adopt proactive and adaptable approaches to crisis management.

Moving forward, it is imperative to employ interdisciplinary perspectives to develop evidence-based strategies for crisis management and resilience building in dental practice. By embracing the lessons learned from the challenges posed by the COVID-19 pandemic, dental professionals can fortify their readiness for future crises. In turn, this helps to sustain the profession's responsibilities, ensuring the continued delivery of care and safeguarding the well-being of both patients and practitioners alike.

## Appendix 1. Interview Guide (Phase One)

NOTE: The following guide outlines questions that were asked within the study interview. Following open-ended qualitative interview methodology, the interviews were tailored to the interests of the interviewee in the general topic, so questions may have been omitted or modified depending on the interviewee. In addition, more specific questions, arising from issues raised in previous interviews or during participant observation, may have been added. However, such changes will be in keeping with the described goals of the study.

### Introduction

This study is looking at the personal lived experiences of dentists during the COVID-19 pandemic in Nova Scotia, assessing the impact that said pandemic has had on the work conditions of this occupational group and if their ability to provide care to patients changed.

Review Letters of Invitation to Participate & Consent with the participant – going over how the data will be used, that names will be kept confidential, any quotations will have identifying factors removed and giving them time to ask questions.

**PART A.** Experience as a dental practitioner / intro into provider background

1. To get started, can you tell me a bit about your background? What is your specific role as a dental practitioner and how long you have been practicing?
2. Can you tell me more about your practice and what setting you work in? How many other providers do you work with? What patient population do you serve?

**PART B.** Working during the COVID-19 pandemic

3. Can you tell me a little bit about what it was like to work during the COVID-19 pandemic?
  - Protocols/ restrictions
  - Outbreaks
  - Personal concerns
  - Access to supplies
  - Financial hardships
  - Workplace culture & Staff attitudes/morale
  - Provincial/federal support & resources
4. What accommodations or modifications have you made to your workplace policies or procedures?
5. What concerns or challenges, if any, have you had during this time related to PPE?
6. Can you tell me about any difficult decisions regarding your practice, staffing, or patient care during this time period?
7. What was your experience with how patient care or the number of patients you saw changed during the pandemic?
  - Patient behaviours and attitudes
  - Challenges/ barriers for specific populations (e.g., elderly)

**PART C.** Overall impact of the COVID-19 pandemic

8. What would you say the overall impact of the pandemic was on work conditions?
9. Can you elaborate a bit on what you think the impact of the pandemic was on patient care?
  - Poor oral health
  - Change in patient-provider interactions or patient health seeking behaviour
10. What was the impact of the pandemic on you as an individual and does this change how you work or think about your profession?
  - Have you ever felt unable to perform your job due to the pandemic or related factors?
11. What lessons have you learned from the pandemic regarding provision of care?
  - Lasting effects on the dental profession
  - Changes in the case of a future pandemic outbreak
12. Is there anything else you would like to share?

Thank you for your participation in this study.
